# Promotion of ROS-mediated apoptosis, G2/M arrest, and autophagy by naringenin in non-small cell lung cancer

**DOI:** 10.7150/ijbs.85443

**Published:** 2024-01-21

**Authors:** Tsung-Ming Chang, Miao-Ching Chi, Yao-Chang Chiang, Chieh-Mo Lin, Mei-Ling Fang, Chiang-Wen Lee, Ju-Fang Liu, Yu Ru Kou

**Affiliations:** 1 School of Dental Technology, College of Oral Medicine, Taipei Medical University, Taipei 11031, Taiwan.; 2 Department and Institute of Physiology, College of Medicine, National Yang-Ming Chiao Tung University, Taipei 11221, Taiwan.; 3 Department of Nursing, Division of Basic Medical Sciences, and Chronic Diseases and Health Promotion Research Center, Chang Gung University of Science and Technology, Chiayi 61363, Taiwan.; 4 Department of Safety Health and Environmental Engineering, Ming Chi University of Technology, New Taipei City 24301, Taiwan.; 5 Division of Pulmonary and Critical Care Medicine, Chang Gung Memorial Hospital, Chiayi 61363, Taiwan.; 6 Department of Respiratory Care, Chang Gung University of Science and Technology, Chiayi 61363, Taiwan.; 7 Research Center for Industry of Human Ecology and Research Center for Chinese Herbal Medicine, Chang Gung University of Science and Technology, Taoyuan 33303, Taiwan.; 8 Graduate Institute of Clinical Medical Sciences, College of Medicine, Chang Gung University, Taoyuan 33302, Taiwan.; 9 Center for Environmental Toxin and Emerging-Contaminant Research, Cheng Shiu University, Kaohsiung 83347, Taiwan.; 10 Super Micro Research and Technology Center, Cheng Shiu University, Kaohsiung 83347, Taiwan.; 11 Department of Orthopaedic Surgery, Chang Gung Memorial Hospital, Chiayi 61363, Taiwan.; 12 School of Oral Hygiene, College of Oral Medicine, Taipei Medical University, Taipei 11031, Taiwan.; 13 Translational Medicine Center, Shin-Kong Wu Ho-Su Memorial Hospital, Taipei 11101, Taiwan.; 14 Department of Medical Research, China Medical University Hospital, China Medical University, Taichung 40402, Taiwan.; 15 Department of Medical Research, Hualien Tzu Chi Hospital, Buddhist Tzu Chi Medical Foundation, Hualien 97002, Taiwan.

**Keywords:** human lung cancer, naringenin, apoptosis, ROS, autophagy

## Abstract

**Background:** As lung cancer is the leading cause of cancer death worldwide, the development of new medicines is a crucial endeavor. Naringenin, a flavanone derivative, possesses anti-cancer and anti-inflammatory properties and has been reported to have cytotoxic effects on various cancer cells. The current study investigated the underlying molecular mechanism by which naringenin induces cell death in lung cancer.

**Methods:** The expression of apoptosis, cell cycle arrest, and autophagy markers in H1299 and A459 lung cancer cells was evaluated using a terminal deoxynucleotidyl transferase dUTP nick end labeling assay (TUNEL), Western blot, Annexin V/PI stain, PI stain, acridine orange staining, and transmission electron microscopy (TEM). Using fluorescence microscopy, DALGreen was used to observe the degradation of p62, a GFP-LC3 plasmid was used to evaluate puncta formation, and a pcDNA3-GFP-LC3-RFP-LC3ΔG plasmid was used to evaluate autophagy flux. Furthermore, the anti-cancer effect of naringenin was evaluated in a subcutaneous H1299 cell xenograft model.

**Results:** Naringenin treatment of lung cancer cells (H1299 and A459) reduced cell viability and induced cell cycle arrest. Pretreatment of cells with ROS scavengers (*N*-acetylcysteine or catalase) suppressed the naringenin-induced cleavage of apoptotic protein and restored cyclin-dependent kinase activity. Naringenin also triggered autophagy by mediating ROS generation, thereby activating AMP-activated protein kinase (AMPK) signaling. ROS inhibition not only inhibited naringenin-induced autophagic puncta formation but also decreased the ratio of microtubule-associated proteins 1A/1B light chain 3 II (LC3II)/LC3I and activity of the AMPK signaling pathway. Furthermore, naringenin suppressed tumor growth and promoted apoptosis in the xenograft mouse model.

**Conclusion:** This study demonstrated the potent anti-cancer effects of naringenin on lung cancer cells, thereby providing valuable insights for developing small-molecule drugs that can induce cell cycle arrest, apoptosis, and autophagic cell death.

## Introduction

Lung cancer is the most common form of cancer 1, 2. The two types of lung cancer, namely small cell (SCLC, 20%) and non-small cell (NSCLC, 80%), differ in terms of symptoms and treatments 3. Treatment options for NSCLC largely depend on cancer stage, cancer cell subtype, and patient's physical condition 4. Whether adjuvant or neoadjuvant therapy is administered, chemotherapy remains a standard treatment for almost all NSCLC patients. However, the cytotoxicity of chemotherapy medicines on normal cells reduces their clinical beneficial effects 5, 6. Thus, there is a pressing need to develop new anti-cancer medication.

Small molecule compounds targeting programmed cell death in human cancers have rapidly progressed 7. Programmed cell death is a process in which cells following stimulation regulate spontaneous as well as programmed death through a series of signaling pathways to maintain homeostasis, such as autophagy-dependent cell death and apoptosis 8-11. Autophagy involves the formation of autolysosomes to maintain cellular homeostasis in response to stress. However, in times of excessive damage, cells initiate programmed autophagic cell death 12, 13. In cancer progression, autophagy plays a dual role: conferring tolerance to adverse growth and enhancing chemotherapeutic resistance 14, 15. In contrast, various natural extracts have shown anti-cancer effects by inducing autophagic death in cancer cells 16-18. These effects of which in inducing apoptosis and autophagy are more pronounced in oral and liver cancer cells 19-21. Therefore, it is crucial to determine whether candidate anti-cancer compounds that induce apoptosis also simultaneously trigger autophagic death.

Naringenin is a citrus flavonoid with anti-inflammatory and anti-viral properties. Previous studies have demonstrated its potential in addressing several lung diseases, including chronic obstructive pulmonary disease, asthma, and coronavirus disease 2019 22-24. Beyond its impact on respiratory conditions, naringenin exhibits remarkable anti-cancer effects, marked by inhibiting cancer cell proliferation and promoting cellular apoptosis 25-27. Naringenin has been shown to suppress the progression of gastric cancer cells by upregulating apoptosis-related proteins, Bax and cleaved caspase-3, while reducing Bcl-2 27. In breast cancer cells, naringenin arrests the cell cycle in the G2/M phase, thereby inhibiting cancer cell proliferation and promoting apoptosis 28. Importantly, studies have elucidated the potential of naringenin in lung cancer, including reducing cellular oxidation and decreasing the initiation and metastasis of lung cancer cells 29-32. Moreover, naringenin can exert its anti-cancer potential by increasing oxidative stress. In pancreatic cancer and placental choriocarcinoma, naringenin induces apoptosis by generating reactive oxygen species (ROS) 27, 33-35. Naringenin also promotes ROS-induced cell cycle arrest in epidermoid carcinoma cells 36. In addition, naringenin protects against palmitate-induced umbilical vein endothelial cell damage and β-amyloid-induced neurotoxicity by activating autophagy 37, 38. Despite numerous studies reporting the anti-cancer activity of naringenin, the relationship between naringenin-induced autophagy and apoptosis in lung cancer has yet to be elucidated.

Our findings in the current study indicate that naringenin promoted cell cycle arrest and apoptosis in lung cancer cells. It also increased acidic organelles, autolysosomes, and autophagic cell death through the AMP-activated protein kinase (AMPK)/mammalian target on the rapamycin (mTOR) signaling pathway. Finally, we confirmed that ROS-mediated naringenin-induced apoptosis and autophagy in NSCLC.

## Materials and Methods

### Chemicals

Primary antibodies specific to mTOR were purchased from Abcam (Cambridge, MA, USA). Akt, phospho-mTOR (Ser2448), phospho-Akt (Ser473), phospho-AMPKα (Thr172), AMPKα, cleaved caspase 3, and cleaved caspase 9 (Cell Signaling; Danvers, MA, USA); Bcl-XL, Bcl-2, Bax, Bak, poly ADP-ribose polymerase (PARP), p62, microtubule-associated proteins 1A/1B light chain 3B (LC3B), and GAPDH (Genetex; Irvine, CA, USA); and cyclin-dependent kinase 1 (CDK1) and cyclin B1 (Merck Millipore; Burlington, MA, USA). Anti-rabbit polyclonal and anti-mouse monoclonal antibodies were purchased from Santa Cruz Biotechnology (Dallas, Texas, USA). ROS scavengers* N*-acetylcysteine (NAC) and H_2_O_2_-scavenging enzyme catalase were purchased from Merck Millipore (Burlington, MA, USA). Pan-caspase inhibitor z-VAD-FMK was purchased from R&D Systems (Minneapolis, MN, USA). Naringenin (N5893) and all other chemicals were purchased from Sigma-Aldrich (St. Louis, MO, USA).

### Cell culture

Cell lines used in this study were as follows: H1299 p53-null human NSCLC cells (American Type Culture Collection; Manassas, VA, USA), A549 p53 wildtype human NSCLC cells, and MRC-5 human fetal normal lung fibroblasts (Bioresource Collection and Research Center; Hsinchu, Taiwan). The cells were maintained according to the recommendations from the suppliers: H1299 (Roswell Park Memorial Institute 1640 medium), A549 (Ham's F-12 nutrient mixture), and MRC-5 (Eagle's minimum essential medium). All cell lines were supplemented with 10% fetal bovine serum (FBS), 100 U/mL penicillin, and 100 μg/mL streptomycin and incubated at 37° C under 5% CO_2_ in air.

### Cell viability assay

Cell viability was assessed using a cell counting kit-8 (CCK-8; Sigma-Aldrich, St. Louis, MO, USA). Cells (1×10^4^) were seeded in 48-well plates and treated with naringenin (25-500 μM) or pretreated with ROS scavengers (catalase, 50 U/mL; NAC, 1 mM), autophagy inhibitors (bafilomycin A1, 100 nM; chloroquine, 50 μM; 3-methyladenine, 100 μM), or a pan-caspase inhibitor (z-VAD-FMK, 20 μM) for 1 h, and then treated with naringenin (250 μM) for another 24 h. Subsequently, the cells were incubated in media with CCK-8 solution (10 μL/well) at 37°C for 4 h. The optical density was measured using a spectrophotometer at 450 nm (BioTek, Winooski, VT, USA).

### Colony formation assay

Cells (1.5×10^5^) were seeded in 6-well plates with a medium containing naringenin (25-500 μM). After 6 h, cells (1×10^3^) were transferred to a new 6-well plate to undergo culturing in naringenin-free medium containing 10% FBS. The culture medium was changed at intervals of three days for 14 days, after which the cells were fixed and stained with 0.05% crystal violet solution. Colony-forming cells were then imaged. Subsequently, an acetic acid solution (33% v/v) was added to the plates, and the absorbance was measured at 550 nm using a microplate reader.

### Apoptosis assay

An Annexin V/PI (Sigma-Aldrich, St. Louis, MO, USA) assay was used to identify apoptotic and necrotic cells following incubation with naringenin (100-500 μM) for 24 h. Following the manufacturer's instructions, live cells were harvested and stained (1 μg/mL PI and 0.025 μg/mL FITC-conjugated annexin V) for 15 min in the dark at room temperature, before analysis using a flow cytometer (Accuri C5, BD, East Rutherford, NJ, USA). In the resulting dot plots, the X-axis represents the intensity of green fluorescence (Annexin V) and the Y-axis represents the intensity of red fluorescence (PI). Cell population distribution analysis was divided into four quadrants: cells that were Annexin V-/PI-, Annexin V+/PI-, Annexin V-/PI+, and Annexin V+/PI+, respectively, represented normal state, early apoptosis, necrosis, and late apoptosis.

### DNA fragmentation and chromatin condensation analysis

DNA damage and chromatin condensation were monitored using 4,6-diamidino-2-phenylindol staining (DAPI; Merck Millipore, Burlington, MA, USA) and terminal deoxynucleotidyl transferase dUTP nick end labeling assays (TUNEL; BD Biosciences Clontech, Palo Alto, CA, USA). Cells were incubated with naringenin (25-500 μM) for 24 h. After fixation and permeabilization, cells were incubated with a TUNEL reaction mixture, and DNA strand breaks were observed using a Nikon Eclipse Ti fluorescence microscope (Nikon, Tokyo, Japan). In chromatin condensation analysis, fixed cells were incubated with DAPI solution (1 μg/ml) for 5 min, and the nucleus morphology was observed using a fluorescence microscope.

### ROS production analysis

Intracellular ROS production was detected via flow cytometry using H_2_DCFDA (Thermo Fisher, Waltham, MA, USA) after treating cells (5×10^5^) to naringenin at various concentrations with H_2_DCFDA at a concentration of 1 μM at 37°C for 30 min. And then, live cells were harvested and removed residual media. In the resulting histogram plots, the X-axis represents the intensity of green fluorescence (2', 7'-DCF) and the Y-axis represents cell counts. The area under the curve is the mean green fluorescence value representing intracellular ROS production of the cells.

### Mitochondria membrane potential

Mitochondrial membrane potential (MMP) and permeability transition, both of which are related to apoptosis, were monitored using JC-1 dye (Thermo Fisher, Waltham, MA, USA). Cells treated with naringenin (25-500 μM) for 24 h were incubated with media containing JC-1 (5 μg/mL) for 30 min. Images were acquired using a fluorescence microscope. The red/green fluorescence ratio was measured respectively at Ex/Em 585/590 nm and Ex/Em 514/529 nm using a microplate reader (Varioskan LUX; Thermo Fisher, Waltham, MA, USA).

### Immunoblotting analysis

Proteins were resolved using sodium dodecyl sulfate-polyacrylamide gel electrophoresis and transferred to immobilon polyvinyldifluoride membranes (Merck Millipore, Burlington, MA, USA). The membranes were blocked and probed using primary antibodies (1:1000) at 4°C overnight. The blots were then incubated with anti-rabbit peroxidase-conjugated secondary antibodies (1:10000) at room temperature for 1 h, after which signals were detected using enhanced chemiluminescence and visualized with UVP chemiluminescence detection system (Analytik Jena US, CA, USA).

### Cell cycle analysis

Cells (5×10^5^) were incubated in 6-well plates with naringenin (25-500 μM) for 24 h. Cells were harvested and fixed in ethanol at -20 °C for 2 h. After removing ethanol, cells were stained using a propidium iodide (PI) solution (100 μg/mL; Sigma-Aldrich, St. Louis, MO, USA), followed by flow cytometry. In the resulting histogram, the X-axis represents PI fluorescence intensity and the Y-axis represents the number of cells.

### Detection of vesicle formation

Cells (5×10^5^) treated with naringenin (25-500 μM) for 24 h were imaged, and the number of cells with vesicles was counted. Data is presented as a percentage of the number of cells containing vesicles and the total number of cells.

### Detection of acidic vesicular organelles

The formation of acidic vacuoles (a hallmark of autophagy) in NSCLC cells was detected using acridine orange staining (1 µg/mL; Sigma-Aldrich, St. Louis, MO, USA). AO emits red fluorescence when excited in an acidic environment and is used to detect the formation of acidic vesicles. Following treatment with naringenin, viable cells were stained with AO for 30 min and the development of acidic organelles was examined using a fluorescence microscope.

### Transmission electron microscopy

H1299 cells (5×10^5^) treated with naringenin (250 μM) for 6 h were first washed to remove the medium and trypsin, after which the suspended cells were immediately fixed in 70% Karnovsky fixative at 4°C until embedding. The cells were then observed under a JEOL JEM-1400 transmission electron microscope (Tokyo, Japan).

### DALGreen autophagy detection

Cells (4×10^4^) were cultured in a 6-well plate and incubated with DALGreen solution (1 μM; Dojindo, Kumamoto, JP) for 30 min, washed twice with culture medium, and then cultured in medium with or without naringenin (250 μM) for 6 h. DALGreen binding to autolysosomes was observed using a fluorescence microscope.

### Autophagy sensor p62-GFP assay

Adherent cells (4×10^4^) were incubated in 6-well plates with 12 μL of p62-GFP reagent (1×10^8^ Premo^TM^ particles/mL; Thermo Fisher, Waltham, MA, USA) for at least 16 h. The cells were then incubated in medium with or without 250 μM naringenin for 6 h. p62-GFP was detected using a fluorescent microscope.

### GFP-LC3 puncta formation and autophagic flux assay

Autophagic activity was assessed in terms of GFP-LC3 fluorescence intensity, GFP-LC3 quench, and GFP-LC3 puncta formation. To measure autophagic flux via GFP-LC3 fluorescence intensity and quench, cells were transfected with GFP-LC3 plasmid or pcDNA3-GFP-LC3-RFP-LC3ΔG (Addgene, MA, USA) using Lipofectamine 3000 transfection reagent (Thermo Fisher, Waltham, MA, USA) for 24 h. Transfected cells (5×10^5^) were then pretreated with or without the autophagy inhibitor bafilomycin A1, followed by incubation in media containing naringenin (250 μM). Fluorescence intensity was evaluated using flow cytometry and a fluorescence microscope. The fluorescence intensity of GFP-LC3 was normalized to RFP-LC3ΔG, and the percentage of each group relative to the control group was quantified. To observe puncta formation, transfected cells (5×10^5^) were pretreated with ROS inhibitors prior to incubation in medium with or without naringenin (250 μM). Fluorescence intensity was evaluated using a fluorescence microscope. Puncta formation is presented as the percentage of the number of cells with puncta and the total number of cells.

### Xenograft assay

All animal experiments were performed following a protocol approved by the Institutional Animal Care and Use Committees of Shin‐Kong Wu Ho‐Su Memorial Hospital (Taipei, Taiwan; IACUC Approval No: 111MOST007). Male 4-week-old Nu/Nu mice were purchased from Lasco (BioLASCO Co., Ltd., Taipei, Taiwan) and housed under pathogen‐free conditions. H1299 cells (2×10^6^ cells in 100 μL) were subcutaneously injected into the dorsal region of the mice. Tumors were allowed to develop until they reached approximately 100 mm^3^ before naringenin treatment was initiated. Mice were randomly assigned to groups of 10. We referred to studies by Qin et al. and Zhang et al. on the anti-cancer effect of naringenin in our selection of doses of 100 mg/kg to 200 mg/kg *in vivo* for experiments 39, 40. In our study, mice were administered a vehicle or naringenin (100 mg/kg) daily for 21 days (10 mice/group) to evaluate the inhibitory effects on tumor growth 39, 40. The volume of the implanted tumor on the dorsal side of the mice was measured every three days using a caliper based on the following formula: *V* = (*L* × *W*^2^)/2, where *V* is the volume (mm3), *L* is the largest diameter (mm), and W is the smallest diameter (mm). After 21 days, the mice were sacrificed via CO_2_ inhalation.

### Immunohistochemistry analysis

Tissue sections (3 μm) were prepared from paraffin-embedded tissues, deparaffinized in xylene, rehydrated in a series of graded alcohols, and washed in deionized water. After performing antigen retrieval, the Novolink^TM^ polymer detection system (Leica, Wetzlar, Germany) was used to conduct IHC assay according to the manufacturer's instructions. Tissue sections were incubated overnight at 4°C with a primary antibody specific for cleaved caspase 3 (1:200) and then washed with PBST. Novolink Polymer was applied at room temperature for 1 h. The stained sections were detected using 3,3′-diaminobenzidine tetrahydrochloride, counterstained with hematoxylin, and observed under a light microscope. The H score was calculated as the sum of the signal intensity (from 0 to 3) multiplied by the percentage of the total area and multiplied by 100. H scores were independently interpreted by three investigators.

### Statistical analysis

SigmaStat 3.5 statistical software was used for processing experimental data. Values were reported as mean ± standard deviation (SD). Statistical comparisons between two samples were performed using the two-tailed test. In contrast, comparisons among more than two groups were performed using a one-way ANOVA with Fisher-LSD post-hoc test. In all cases, *p* ≤ 0.05 was considered significant.

## Results

### Naringenin suppressed cancer cell proliferation and induced cell apoptosis

**Figure 1:**
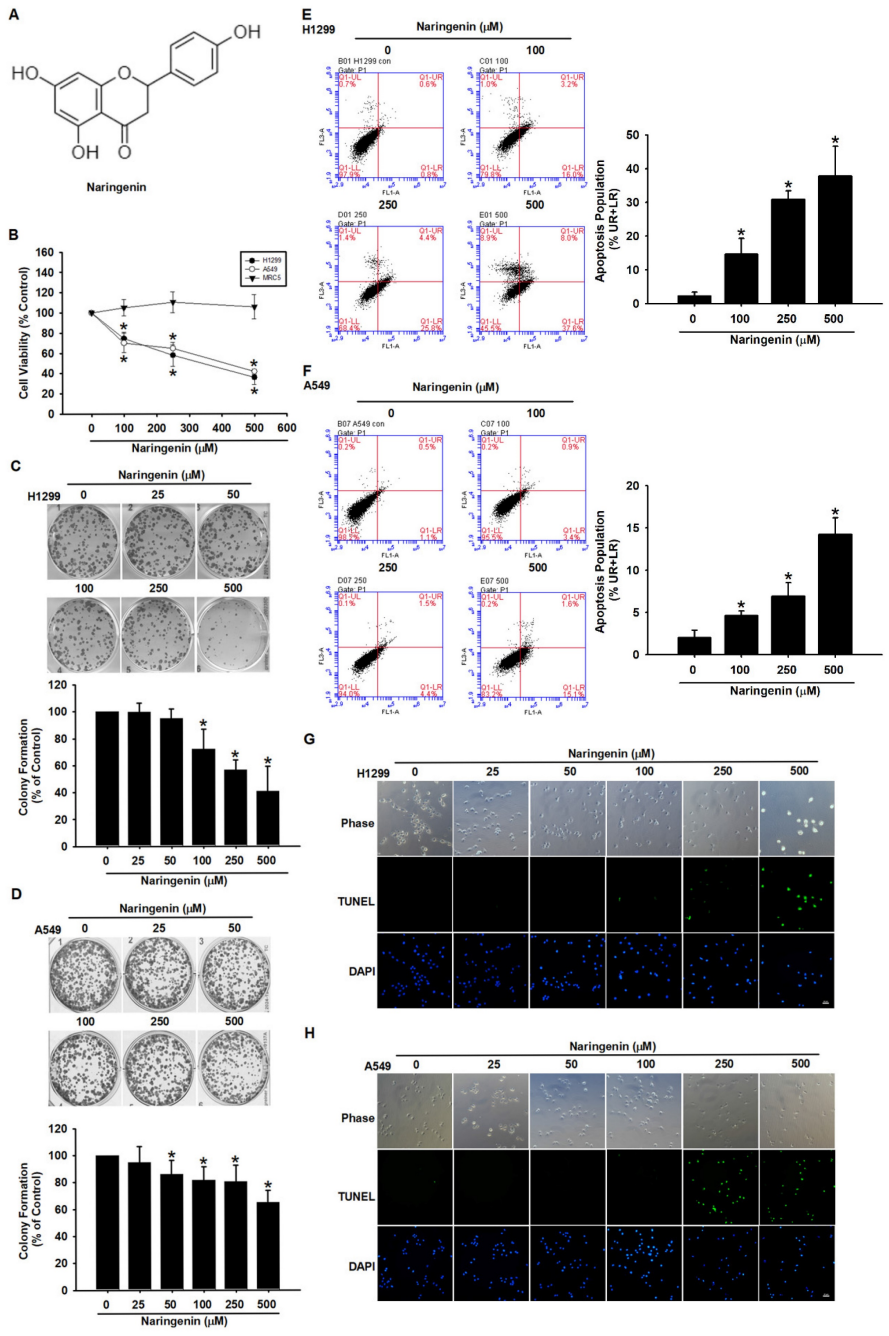
** Naringenin suppressed proliferation and induced apoptosis in non-small cell lung cancer cells.** (**A**) Chemical structure of naringenin. (**B**) MRC-5, H1299, and A549 cells were treated with naringenin (100-500 μM) for 24 h, after which cell viability was assessed by CCK-8 assay (n=4). (**C**) H1299 and (**D**) A549 cells were treated with naringenin (25-500 μM) for 6 h and then incubated in naringenin-free medium for a further 14 days, after which cell reproductive viability was assessed by colony formation assay (n=4). (**E**) H1299 and (**F**) A549 cells were treated with naringenin (100-500 μM) for 24 h and then stained with Annexin V/PI; cell death was analyzed by flow cytometry (n=4). (**G**) H1299 and (**H**) A549 cells were treated with naringenin (25-500 μM) for 24 h, and then visualized by optical microscopy. DNA fragmentation and chromatin condensation were respectively examined by TUNEL assay and DAPI staining via fluorescence microscopy. Untreated cells were used as control. Scale bar = 20 μm. Results are shown as means ± SD. *p < 0.05 compared with untreated control.

We first examined the cytotoxic effects of naringenin (Figure 1A) in normal lung fibroblasts (MRC-5) and lung cancer cells (H1299 and A549) using cell viability assay and cell proliferation assay. We found that naringenin significantly decreased the survival rates of H1299 and A549 cells in a dose-dependent manner, with respective IC_50_ values of 330.7 μM and 400.4 μM (Figure 1B); note that the viability of MRC-5 cells was not affected. In colony formation assays, naringenin significantly suppressed cancer cell proliferation in both NSCLC cell lines, but it did not in MRC-5 cells (Figure 1C-D and S1). These results suggest that naringenin possessed anti-cancer effects on lung cancer cells without noticeable adverse effects on normal cells.

The mechanism underlying naringenin-induced cell death was explored using Annexin V/PI double staining. Annexin V binds to phosphatidylserine, which is exposed on the outer membrane during early apoptosis, whereas PI stains the DNA of dead cells. The percentage of apoptotic H1299 and A549 cells significantly increased after incubation with naringenin for 24 h (H1299: 100 μM, 14.6±4.71%; 250 μM, 30.9±2.60%; 500 μM, 37.8±8.81%; and A549: 100 μM, 4.7±0.54%; 250 μM, 7.0±1.59%; 500 μM, 14.2±1.99%) (Figure 1E-F). An examination of the modes of apoptotic cell death using DAPI and TUNEL staining revealed that nuclei fragmentation and chromatin condensation dramatically increased following naringenin treatment (Figure 1G-H). Taken together, these results indicate that naringenin caused non-small cell lung cancer cell death by promoting apoptosis and inhibiting cell proliferation.

### Naringenin promoted apoptosis through ROS production and MMP perturbation

**Figure 2:**
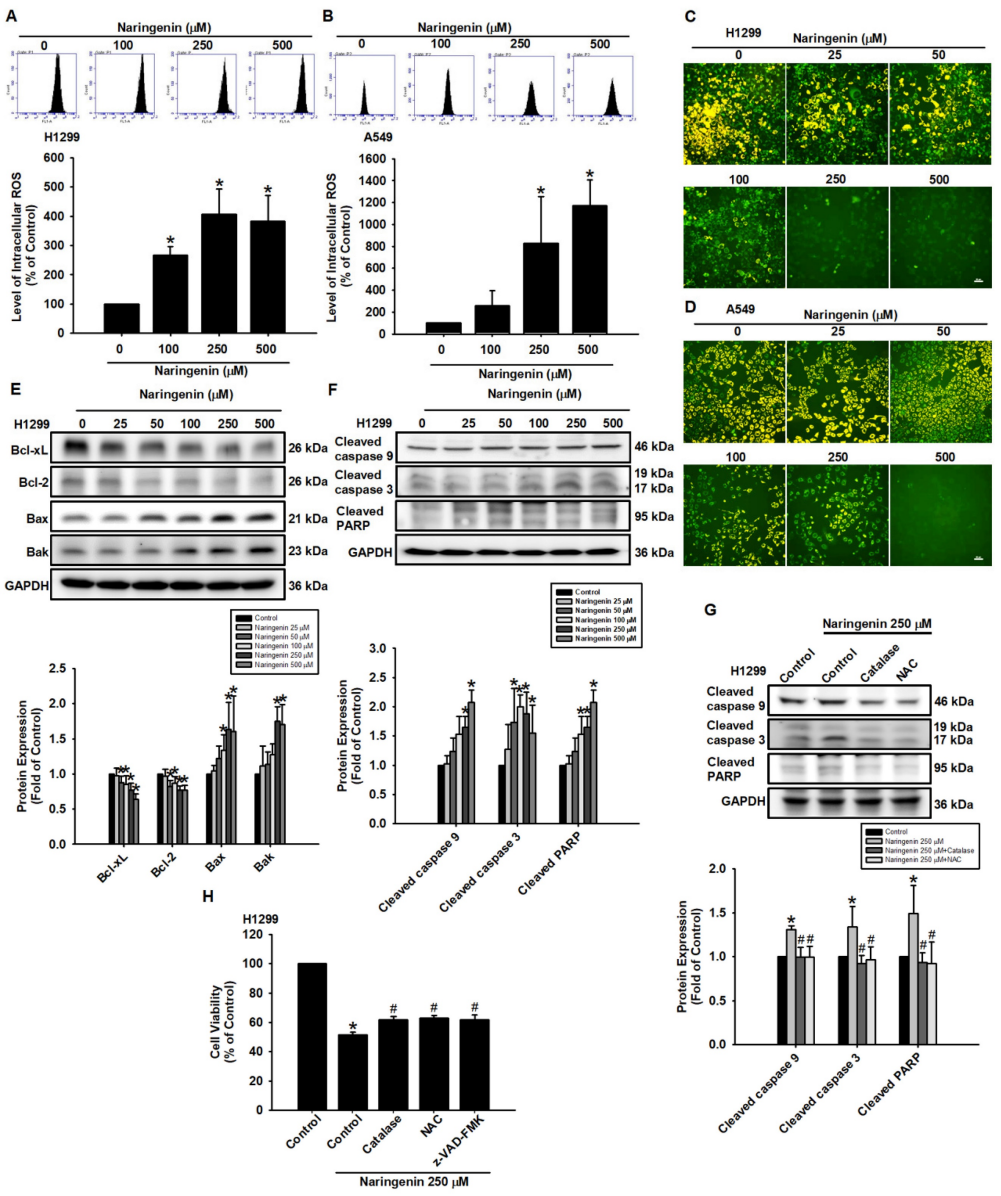
** Naringenin induced intracellular ROS and promoted apoptosis by altering MMP in non-small cell lung cancer cells.** (**A**) H1299 and (**B**) A549 cells were treated with naringenin (100-500 μM) and then inculcated with H_2_DCFDA for 30 min, after which ROS production was evaluated by flow cytometry (n=4). (**C**) H1299 and (**D**) A549 cells were treated with naringenin (25-500 μM) for 24 h and then incubated with JC-1 for 30 min. MMP was observed by fluorescence microscopy (n=4). Scale bar = 20 μm. (**E-F**) H1299 cells were treated with naringenin (25-500 μM) for 8 h; by Western blot, expression levels of (**E**) Bcl-xL, Bcl-2, Bak, and Bax and (**F**) Cleaved caspase 3, cleaved caspase 9, and cleaved PARP were examined (n=4). (**G**) H1299 cells were pretreated with ROS scavengers, catalase (H_2_O_2_-scavenging enzyme, 50 U/mL) or N-acetylcysteine (NAC, 1 mM), for 1 h and then treated with naringenin (250 μM) for 8 h; protein expression levels of cleaved caspase 3, cleaved caspase 9, and cleaved PARP were examined by Western blot (n=4). (**H**) H1299 cells were pretreated with catalase (50 U/mL), NAC (1 mM), or pan-caspase inhibitor z-VAD-FMK (20 μM) for 1 h and then treated with naringenin (250 μM) for 24 h; cell viability was assessed using a CCK-8 assay (n=4). Untreated cells were used as control. Results are shown as means ± SD. *p < 0.05 compared with untreated control. #*p* < 0.05 compared with group treated with naringenin.

The relationship between ROS and apoptosis has been addressed in previous studies 41, 42. In the present study, we sought to further investigate the anti-cancer effects of naringenin by examining ROS production and the mechanism of naringenin-induced cell death in NSCLC cells. Treatment of A549 and H1299 cells with naringenin significantly increased intracellular ROS production levels in a dose-dependent manner (Figure 2A-B) while reducing MMP (Figure 2C-D and S2). The contribution of MMP depolarization on intrinsic apoptosis was explored by examining the expression of the apoptosis-related Bcl-2 family proteins Bcl-2 and Bcl-xL, which are responsible for stabilizing the integrity of the mitochondrial outer membrane 43. Stimulating NSCLC cells with naringenin decreased the expression of Bcl-xL and Bcl-2 while promoting the expression of Bax and Bak proteins, which are known to perforate the mitochondrial outer membrane to initiate apoptosis (Figure 2E and S5A). The loss of MMP led to a corresponding cleavage of caspase 9, caspase 3, and PARP (Figure 2F and S5B). Pretreatment of cells with ROS scavengers NAC and catalase significantly reduced naringenin-induced cleavage of caspase 9, caspase 3, and PARP while slightly but significantly restoring naringenin-mediated cell death (Figure 2G-H). Taken together, these results indicate that naringenin promoted apoptosis in lung cancer cells by inducing ROS.

### Naringenin induced cell cycle arrest via ROS production in NSCLC cells

**Figure 3:**
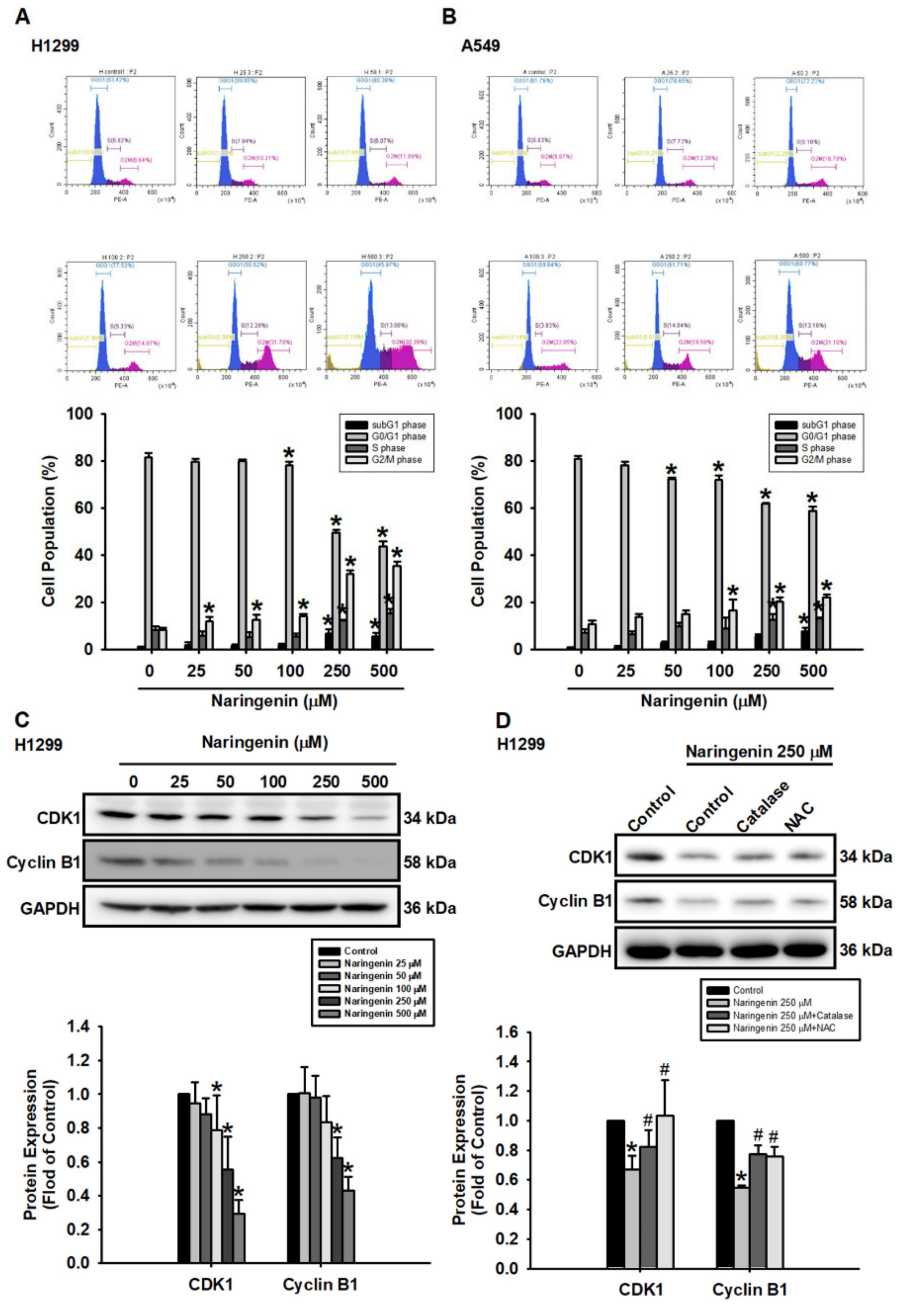
** Naringenin induced cell cycle arrest via ROS production in non-small cell lung cancer cells.** (**A**) H1299 and (**B**) A549 cells were treated with naringenin (25-500 μM) for 24 h and then stained with PI for 30 min; cell cycle was evaluated by flow cytometry (n=4). (**C**) H1299 cells were treated with naringenin (25-500 μM) for 6 h; CDK1 and cyclin B1 protein expression was examined by Western blot (n=4). (**D**) H1299 cells were pretreated with ROS scavengers, catalase (50 U/mL) or NAC (1 mM), for 1 h and then treated with naringenin (250 μM) for 6 h; protein expression levels of CDK1 and cyclin B1 were examined by Western blot (n=4). Untreated cells were used as control. Results are shown as means ± SD. *p < 0.05 compared with untreated control. #*p* < 0.05 compared with group treated with naringenin.

Treating H1299 and A549 cells with naringenin at various concentrations (25-500 μM) for 24 h significantly shifted the distribution of cells from the G0/G1 phase to the subG1 and G2/M phase (Figure 3A and 3B). G2/M phase arrest was associated with the downregulation of CDK1 and cyclin B1 (Figure 3C and S6A), the effects of which were reversed following treatment with ROS scavengers (Figure 3D and S6B). Taken together, these results confirm that the effects of naringenin in reducing cell viability were accompanied by the arrest of the G2/M cell cycle and the downregulated expression of CDK1 and cyclin B1 via ROS production.

### Naringenin promoted autophagy through ROS-induced activation of AMPK in NSCLC cells

**Figure 4:**
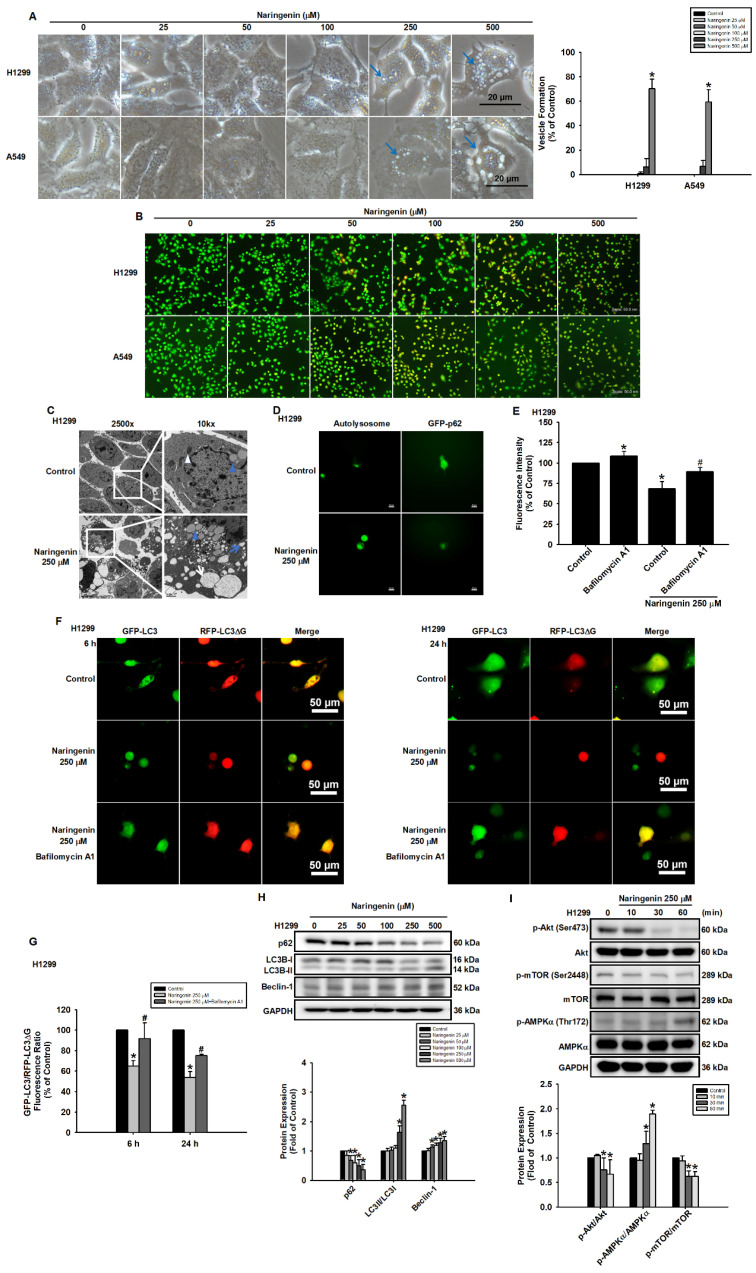
** Naringenin promoted autophagy in non-small cell lung cancer cells.** (**A**) Left: H1299 and A549 cells were treated with naringenin (25-500 μM) for 24 h, after which cell morphology (blue arrows: vesicles) was observed by optical microscopy. Scale bar = 20 μm. Right: Quantitative analysis of vesicle formation (n=4). (**B**) H1299 and A549 cells were treated with naringenin (25-500 μM) for 24 h and then stained with acridine orange for 30 min; acidic organelles were visualized by fluorescence microscopy. Scale bar = 50 μm. (**C**) H1299 cells were treated with naringenin (250 μM) for 6 h, after which autolysosomes (white arrow) were observed by TEM; white triangle indicates mitochondria, blue triangles indicate lysosomes, the blue arrows indicate vacuoles, and the white arrows indicate autolysosomes. 2,500×: Scale bar = 5 μm. 10,000×: Scale bar = 1 μm. (**D**) H1299 cells were incubated with DALGreen solution or p62-GFP reagent and then treated with naringenin for 6 h, after which autolysosomes and GFP-p62 were observed by fluorescence microscopy. Scale bar = 20 μm. (**E**) H1299 cells transfected with GFP-LC3 were pretreated with autophagy inhibitor bafilomycin A1 (100 nM) for 1 h and then treated with naringenin (250 μM) for 6 h; autophagic activity was assessed by flow cytometry (n=4). (**F**) H1299 cells transfected with pcDNA3-GFP-LC3-RFP-LC3ΔG were pretreated with autophagy inhibitor bafilomycin A1 (100 nM) for 1 h and then treated with naringenin (250 μM) for 6 h and 24 h, after which autophagic flux was observed by fluorescence microscopy (n=4). Scale bar = 50 μm. (**G**) Quantitative analysis of GFP-LC3/RFP-LC3△G fluorescence ratio (n=4). (**H**) H1299 cells were treated with naringenin (25-500 μM) for 6 h; protein expression of p62 and Beclin-1 as well as the LC3II/LC3I ratio were examined by Western blot (n=4). (**I**) H1299 cells were treated with naringenin (250 μM) for the indicated times, after which the phosphorylation of Akt, mTOR, and AMPKα was examined by Western blot (n=4). Untreated cells were used as control. Results are shown as means ± SD. *p < 0.05 compared with untreated control. #*p* < 0.05 compared with control treated with naringenin.

Previous studies have reported that naringenin induces autophagy in neural cells and mice 44, 45. This prompted us to examine whether mechanisms associated with autophagy were altered in lung cancer cells in response to naringenin. Microscopy image analysis revealed the presence of vesicles, which are hallmarks of autophagy, following naringenin treatment in H1299 and A549 cells (Figure 4A). Acridine orange staining further revealed the formation of acidic autophagic vesicles, as evidenced by bright red staining (Figure 4B). Following the confirmation of autolysosomes in naringenin-treated H1299 cells by TEM (Figure 4C), fluorescence microscopy image analysis was used to clarify changes in autophagy molecular markers. We found that naringenin induced autolysosome expression and decreased p62 expression (Figure 4D), which indicated substantial autophagy activity. Pretreatment with an autophagy inhibitor (bafilomycin A1) blocked naringenin-induced autolysosome formation and reduced the degradation of GFP-LC3 (Figure 4E and S3). In order to examine autophagy flux, a pcDNA3-GFP-LC3-RFP-LC3ΔG plasmid was used as a fluorescent probe. The cleavage of the C-terminus of LC3 by autophagy-related 4 cysteine peptidase leads to the cleavage of the GFP-LC3-RFP-LC3ΔG tandem protein into GFP-LC3 and RFP-LC3ΔG. GFP-LC3 can then conjugate to phosphatidylethanolamine and is subsequently degraded by autolysosomes, while RFP-LC3ΔG cannot conjugate to phosphatidylethanolamine and remains in the cytosol. This served as an internal control to evaluate autophagic flux by observing a decrease in GFP fluorescence compared to RFP fluorescence. The quenching of green fluorescence following 6 h of naringenin treatment, indicating the degradation of GFP-LC3. This degradation persisted until at least 24 h (Figure 4F). However, pretreatment of H1299 cells with bafilomycin A1 blocked naringenin-induced green fluorescence quenching, providing further evidence that naringenin increased autophagy flux via autolysosome formation (Figure 4F and 4G). Western blot analysis revealed that naringenin reduced the expression of p62 as well as increased the expression of Beclin-1 and the LC3II/LC3I ratio (Figure 4H and S7A). As researchers have speculated that AMPK/Akt/mTOR signaling plays an essential role in autophagy by regulating autolysosome formation 46, we investigated whether AMPK/Akt/mTOR signaling was involved in naringenin-induced autophagy. Compared to the control group, naringenin significantly decreased Akt and mTOR phosphorylation and promoted AMPKα phosphorylation in NSCLC cells (Figure 4I and S7B), indicating that naringenin altered the expression of autophagy-related proteins by activating AMPKα and inactivating the Akt and mTOR signaling pathways. This could explain the production of autolysosomes and the promotion of autophagic activity in NSCLC cells.

**Figure 5:**
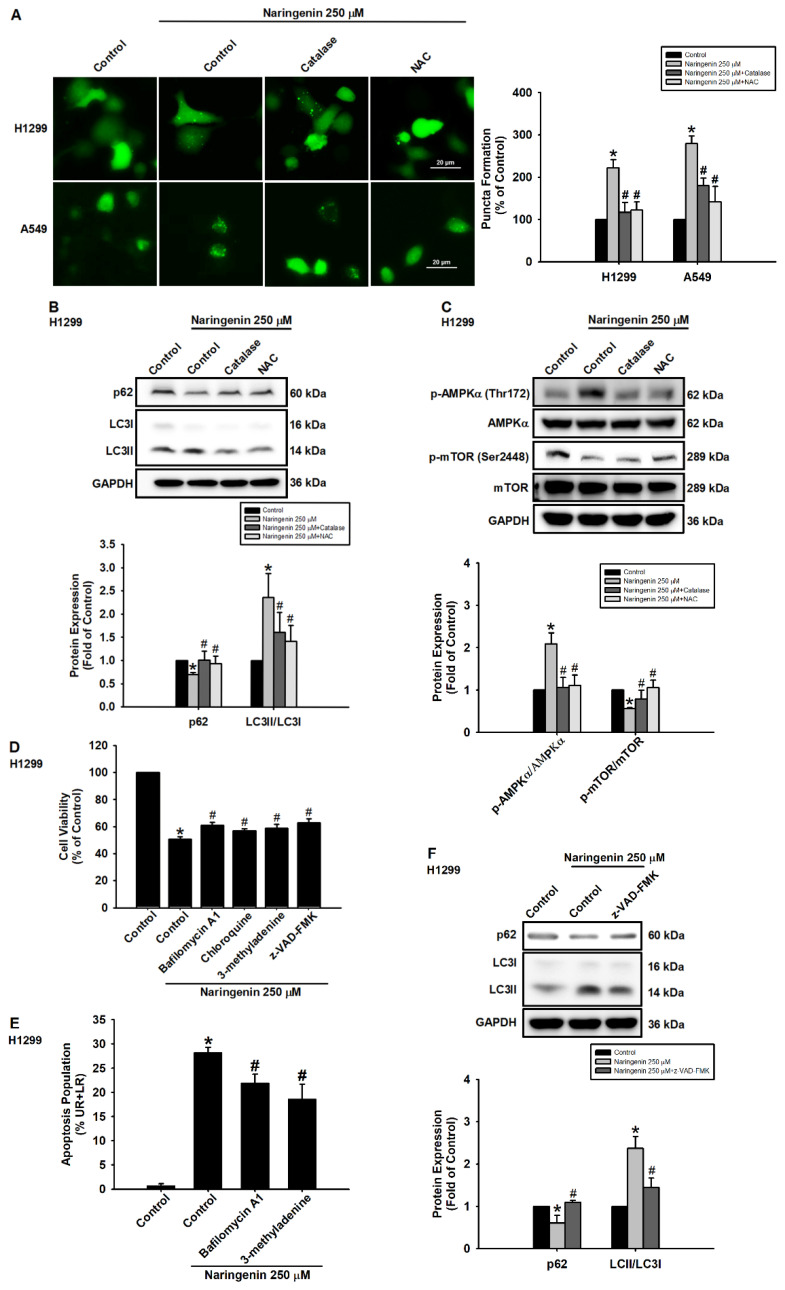
** Naringenin promoted cell death via ROS-induced autophagy.** (**A**) Left: H1299 and A549 cells transfected with GFP-LC3 were pretreated with ROS scavengers, catalase (50 U/mL) or NAC (1 mM), for 1 h and then treated with naringenin (250 μM) for 6 h; puncta formation was observed by fluorescence microscopy. Scale bar = 20 μm. Right: Quantitative analysis of puncta formation (n=4). (**B**) H1299 cells were pretreated with ROS scavengers for 1 h and then treated with naringenin (250 μM) for 6 h; expression of p62 protein and LC3II/LC3I ratio were measured by Western blot (n=4). (**C**) H1299 cells were pretreated with ROS scavengers for 1 h and then treated with naringenin (250 μM) for 1 h; phosphorylation of AMPKα and mTOR was examined by Western blot (n=4). (**D**) H1299 cells were pretreated with autophagy inhibitors bafilomycin A1 (100 nM), chloroquine (50 μM), or 3-methyladenine (100 μM) as well as pan-caspase inhibitor z-VAD-FMK (20 μM) for 1 h and then treated with naringenin (250 μM) for 24 h; cell viability was assessed by CCK-8 assay (n=4). (**E**) H1299 cells were pretreated with bafilomycin A1 (100 nM) and 3-methyladenine (100 μM) for 1 h and then treated with naringenin (250 μM) for 24 h; cell apoptosis was assessed by Annexin V/PI stain (n=4). (**F**) H1299 cells were pretreated with z-VAD-FMK (20 μM) for 1 h and then treated with naringenin (250 μM) for 6 h; expression of p62 and the LC3II/LC3I ratio was measured by Western blot (n=4). Results are shown as means ± SD. *p < 0.05 compared with untreated control. #*p* < 0.05 compared with group treated with naringenin.

The role of ROS in naringenin-induced autophagy via the formation of puncta was explored by transiently transfecting H1299 and A549 cells with a plasmid, GFP-LC3 plasmid and then pretreating the cells with or without ROS inhibitors before naringenin treatment. Pretreatment with ROS scavengers, catalase and NAC, suppressed the naringenin-induced formation of GFP-LC3 puncta (Figure 5A). In NSCLC cells, the ROS inhibitors also induced naringenin-reduced p62 expression, decreased the LC3II/LC3I ratio (Figure 5B and S8A), and reversed naringenin-induced AMPKα phosphorylation and naringenin-suppressed mTOR phosphorylation (Figure 5C and S8B). We next sought to confirm the biological role of naringenin in the regulation of autophagic flux in lung cancer cells. Pretreatment of H1299 cells with bafilomycin A1 (an inhibitor of autophagosome-lysosome fusion), chloroquine (CQ, an inhibitor of autophagosomes/lysosomes), and 3-methyladenine (3-MA, an inhibitor of the PI3 complex and autophagosomes) prior to naringenin treatment was shown to modulate naringenin-induced cell death and improve cell viability (Figure 5D). In addition, 3-methyladenine and bafilomycin A1 significantly inhibited naringenin-induced apoptosis in H1299 cells (Figure 5E), and z-VAD-FMK (a pan-caspase inhibitor) reversed the naringenin-induced changes in autophagy proteins (Figure 5F). These results suggest that naringenin-induced autophagy and apoptosis worked synergistically to regulate cell death and that naringenin triggered ROS production to induce autophagy via AMPK/Akt/mTOR signaling, leading to cell death.

### Naringenin suppressed lung cancer growth in a mouse xenograft model

**Figure 6:**
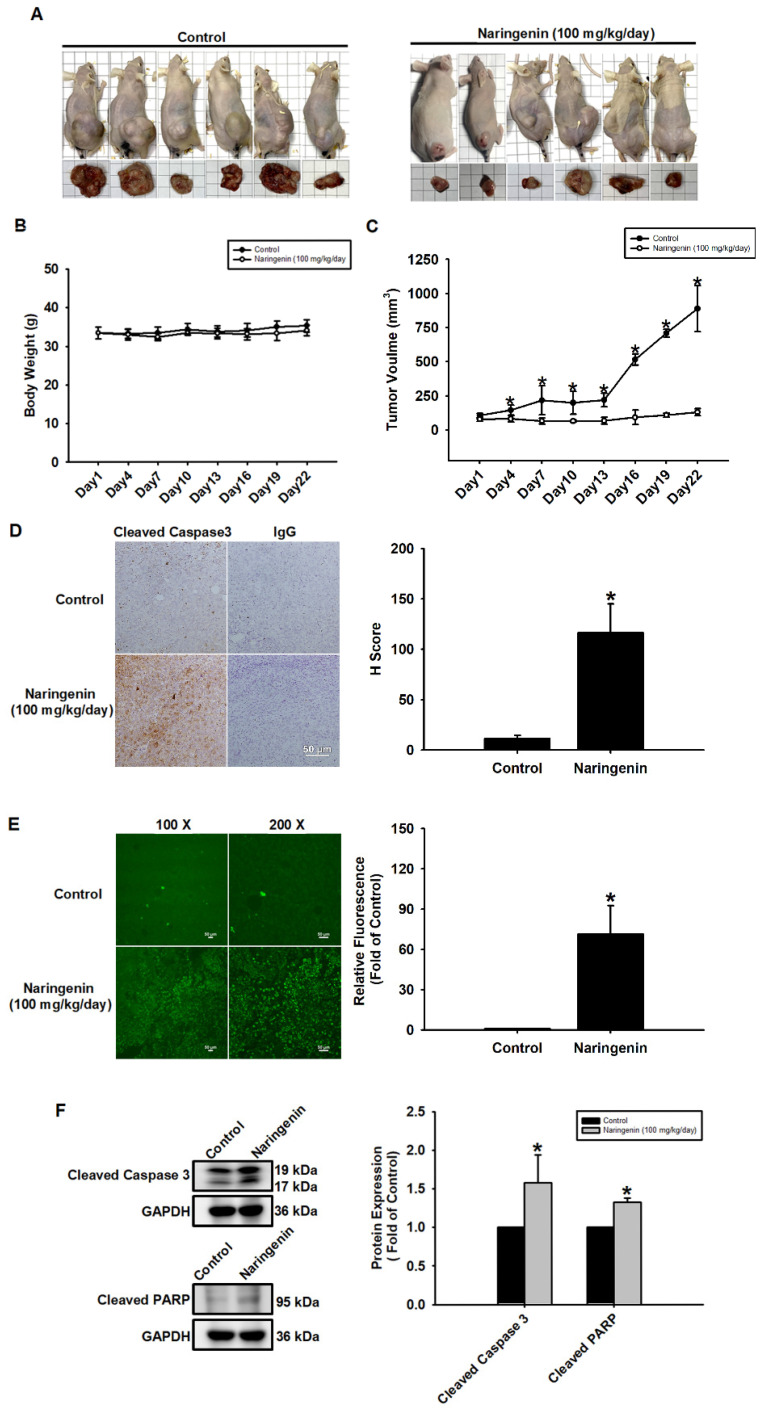
** Naringenin suppressed tumor growth and induced apoptosis *in vivo*.** (**A**) Representative images of mice and tumors. (**B**) Body weight and (**C**) tumor volume of control and naringenin-treated groups (n=6). (**D**) Left: Expression of cleaved caspase 3 in control and naringenin-treated tumor tissues was analyzed by IHC. Scale bar = 20 μm. Right: Quantitative analysis of H score of cleaved caspase3. (**E**) Left: Apoptosis in control and naringenin-treated tumor tissues was analyzed by TUNEL assay. Scale bar = 50 μm. Right: Quantitative analysis of relative fluorescence of TUNEL-positive cells in control and naringenin-treated groups. (**F**) Expression levels of cleaved caspase 3 and cleaved PARP in control and naringenin-treated tumor tissues were examined by Western blot (n=4). Results are shown as means ± SD. *p < 0.05 compared with untreated control.

A subcutaneous xenograft model was used to investigate the therapeutic effects of naringenin on lung cancer and tumor growth. Mice were treated daily with a vehicle or naringenin (100 mg/kg) from the day that tumor volume reached approximately 100 mm^3^. Tumor volume was measured at 3-day intervals using a caliper. Compared to the vehicle-treated control group, naringenin significantly inhibited tumor growth from day 4 (Figure 6A and 6C) with no effects on body weight (Figure 6B). IHC and TUNEL assays of tumor sections revealed that naringenin can promote lung cancer cell apoptosis (Figure 6D and 6E). Western blot analysis of the tumors revealed that the expression of cleaved caspase 3 and cleaved PARP was significantly higher with naringenin treatment (Figure 6F). Taken together, these findings demonstrate the efficacy of naringenin in suppressing tumor growth by promoting apoptosis in H1299 cells *in vivo*.

## Discussion

**Figure 7:**
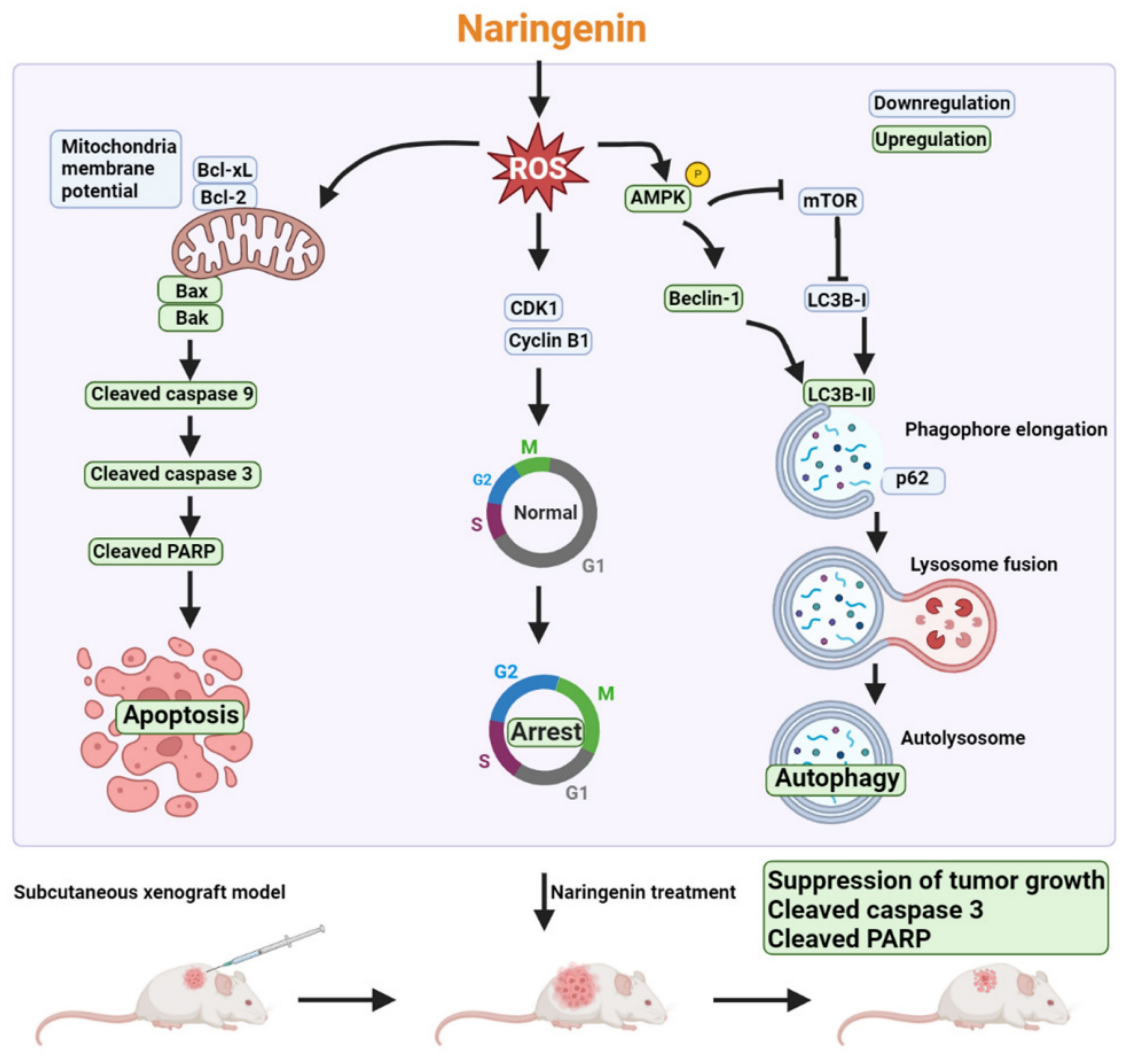
** Schematic mechanism of naringenin promoting apoptosis, cell cycle arrest, and autophagy in lung cancer.** Triggering of intracellular ROS by naringenin reduces mitochondrial membrane potential (MMP) as well as CDK1 and cyclin B1 expression and activates AMPK. Loss of MMP results in a reduction of anti-apoptotic proteins Bcl-xL and Bcl-2 but an increase in pro-apoptotic proteins Bax and Bak to subsequently initiate the caspase 9/3 cascade. Suppression of CDK1 and cyclin B1 promotes cell cycle arrest in the G2/M phase. Naringenin not only induces AMPK activation but also inhibits mTOR phosphorylation to subsequently trigger autophagic cell death by inducing the expression of Beclin-1 and LC3B-II, degradation of p62, and formation of autolysosome. The anti-cancer effect of naringenin was further confirmed in a subcutaneous xenograft lung cancer model.

Despite the numerous treatment strategies available for various stages of NSCLC, lung cancer remains the leading cause of cancer death worldwide 2. Chemotherapy is a standard treatment prescribed to most lung cancer patients; however, the low specificity of chemotherapy drugs lowers their clinical applicability 4. Various natural products have been shown to exhibit anti-cancer activity by promoting apoptosis or autophagy 47, 48. Naringenin has demonstrated anti-cancer or adjunctive effects when combined with chemotherapy, such as the inhibition of cell proliferation and the promotion of apoptosis 49-52. Nonetheless, the mechanisms underlying the anti-cancer effects of naringenin in lung cancer remain unclear. Our results revealed that the potent effects of naringenin on lung cancer can be attributed to promote cell cycle arrest, apoptosis, and autophagy, all of which leads to cell death (Figure 7).

Intracellular ROS production has been shown to induce mitochondrial dysfunction and thus apoptosis 53, 54. The well-documented roles of ROS in promoting cell cycle arrest in many cancers have led to the inclusion of ROS in the design of many anti-cancer drugs 55, 56. In addition, several chemotherapeutic agents, including gemcitabine, trichostatin A, epigallocate-3-gallate, capsaicin, and benzyl isothiocyanate, aim to increase cellular ROS levels in a bid to enhance the apoptosis of tumor cells 57-62. Flavonoids such as naringenin act as antioxidants under normal conditions but as potent pro-oxidants in cancer cells that modulate apoptotic pathways 63, such as the inhibition of glutathione reductase activity 64. In a previous study on human epidermoid cancer cells, naringenin was shown to trigger ROS production and promote cancer cell apoptosis and cell cycle arrest 36. Similar to Liu *et al.* 4*,* we observed a remarkable increase in ROS production following naringenin treatment, resulting in the activation of caspase cascades and the inhibition of cell proliferation (Figure 2G-H). ROS scavengers were also shown to reverse naringenin-induced AMPK signaling and autophagic activity (Figure 5). Our results support previous findings indicating that the effects of naringenin in promoting cell cycle arrest, apoptosis, and autophagy in cancer cells are associated with intracellular ROS production.

During chemotherapy treatment, autophagy acts counterintuitively by maintaining cell homeostasis and promoting cancer cell survival. However, researchers have developed several anti-cancer drugs capable of inducing apoptosis via hyperactive autophagy 65, 66. In many cancers, flavonoids induce autophagy and apoptosis, as well as inhibit cell progression by modulating ROS 63. In the current study, naringenin promoted autophagy by inducing ROS production in NSCLC cells. Pretreatment with autophagy inhibitors restored naringenin-suppressed viability in H1299 cells (Figure 5D). The phosphatidylinositol 3-kinase/Akt/mTOR and AMPK pathways are crucial regulator of autophagy, proliferation, and survival of cancer cells 67, 68. Indeed, we found that naringenin inhibited mTOR phosphorylation and promoted AMPK activation, while pretreatment with ROS scavengers reversed these effects (Figure 5C and S8B). These results suggest that naringenin causes autophagy via the ROS-mediated phosphatidylinositol 3-kinase/Akt/mTOR and AMPK signaling pathway in human NSCLC cells. In Figure 5D, the results further indicated that naringenin causes autophagic cell death.

Interestingly, autophagy inhibitors only slightly but significantly reversed naringenin-induced cell death. The result led us to investigate whether the mechanism of naringenin-induced cell death is related to the interaction between apoptosis and autophagy. Autophagy and apoptosis are inter-connected in the anti-cancer process, exerting synergistic, promotional, and antagonistic effects 69. In addition, autophagic cell death highly depends on the cell type, the source of signals, and the duration of stimulation 70. We observed that autophagy inhibitors and apoptosis inhibitors respectively reduced the number of apoptotic cells and autophagy proteins induced by naringenin, thus confirming that naringenin-induced apoptosis and autophagic cell death synergistically (Figure 5E and 5F). Our findings reveal that naringenin promotes cell death in lung cancer cells due to the lethal synergistic effects of ROS stimulating apoptosis and autophagy.

A growing body of evidence suggests that naringenin suppresses tumor growth in mice with breast cancer, glioblastoma, and skin cancer 39, 71, 72. Previous studies have found that ingesting 600 to 900 mg of naringenin had no significant adverse effects on heart rate or blood pressure. Similarly, no significant adverse effects were observed following the administration of naringenin (up to 100 mM) on rat intestinal epithelial cells, indicating a comparable safety profile *in vivo* and* in vitro* studies 73, 74. In the current study, naringenin significantly decreased tumor volume *in vivo* and promoted tumor apoptosis. The fact that there was no indication of naringenin cytotoxicity in healthy lung cells and no adverse effects on body weight further supports naringenin as a potent candidate compound in the development of novel cancer treatments (Figure 1B, 6B, and S1).

The reason for this result may be due to the mechanisms of naringenin activity involved the downregulation of CDKs, cyclins, and anti-apoptotic proteins with a corresponding activation of pro-apoptotic proteins in cancer cells. Note that the levels of CDKs and cyclins in normal cells remained at basal levels, i.e., naringenin did not affect cell cycle activity at basal levels. Our findings suggest that in NSCLC cells, naringenin suppressed the expression of CDK1, cyclin B1, and anti-apoptotic proteins as well as upregulated pro-apoptotic pathways. This evidence underscores the pivotal role of naringenin as a promising and versatile candidate in the development of innovative therapeutic strategies for non-small cell lung cancer.

## Conclusion

This study confirms the mechanisms of naringenin in inhibiting the proliferation of NSCLC cells and inducing cell death both *in vitro* and *in vivo*. Our results have further elucidated the potent anti-cancer effects of naringenin in non-small cell lung cancer and provide strategies for developing small-molecule compounds that induce cell cycle arrest, apoptosis, and autophagy through enhanced ROS production.

## Supplementary Material

Supplementary figures.
