# Supplementary material for: Promotion of ROS-mediated apoptosis, G2/M arrest, and autophagy by naringenin in non-small cell lung cancer
Source: Int J Biol Sci. 2024 Jan 21;20(3):1093–109. doi: 10.7150/ijbs.85443 (PMC10845293; doi:10.7150/ijbs.85443)
Supplement: Supplementary file 1 — Supplementary figures. [file ijbsv20p1093s1.pdf]

**Supplemental figures and legends**

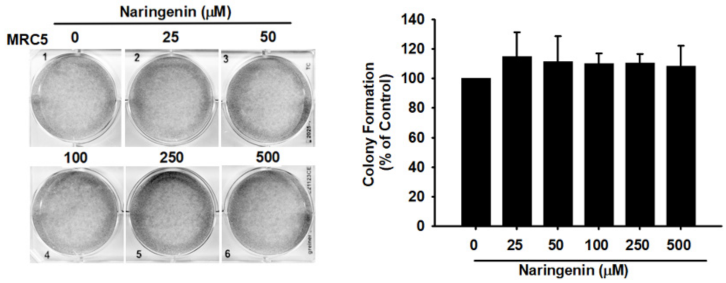


**Figure S1. Cytotoxic effect of naringenin in normal cells.** MRC5 cells were treated with naringenin (25-500 μM) for 6 h and then incubated for a further 14 days in naringenin-free medium. Cell reproductive viability was assessed by colony formation assay (n=4). Untreated cells were used as control. Results are shown as means ± SD.

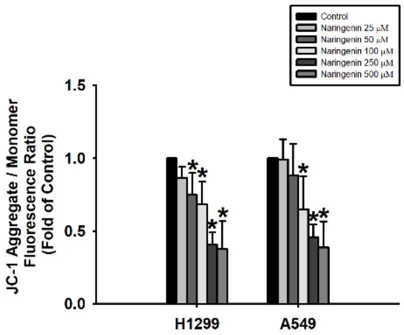


**Figure S2. Naringenin promoted perturbations in mitochondrial membrane potential in non-small cell lung cancer cells.** H1299 and A549 cells were treated with naringenin (25-500 μM) for 24 h and then incubated with JC-1 for 30 min; fluorescence intensity was measured by a fluorescence plate reader (n=4). Untreated cells were used as control. Results are shown as means ± SD. *$p < 0.05$ compared with untreated control.

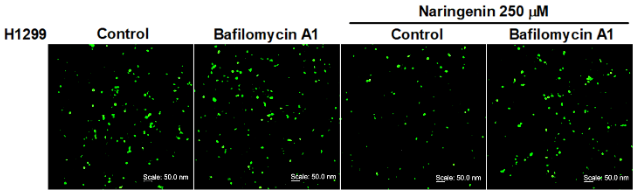


**Figure S3. Naringenin promoted autophagic flux in non-small cell lung cancer cells.** GFP-LC3-transfected H1299 cells were pretreated with autophagy inhibitor bafilomycin A1 (100 nM) for 1 h, followed by treatment with naringenin (250 μM) for 6 h. Autophagic flux was assessed by fluorescent microscopy (n=4). Scale bar = 50 nm. Untreated cells were used as control.

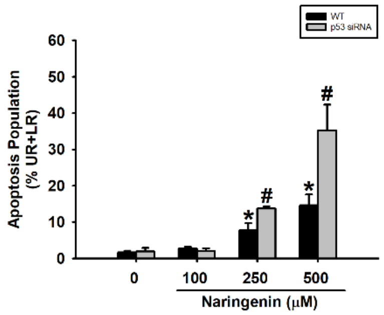


**Figure S4. Transfection of p53 siRNA increased naringenin-induced apoptosis in A549 cells.** WT and p53 siRNA-transfected A549 cells were treated with naringenin (100-500 μM) for 24 h. Apoptosis was assessed by Annexin V/PI assay (n=4). Untreated cells were used as controls. Results are shown as means ± SD. *$p < 0.05$ compared with untreated control. #$p < 0.05$ compared with WT cells.

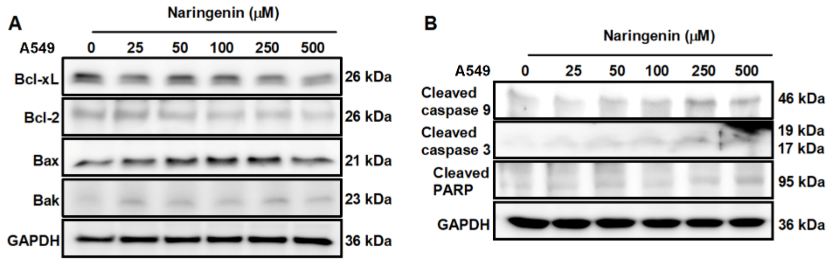


**Figure S5. Naringenin promoted apoptosis in A549 cells.** A549 cells were treated with naringenin (25-500 μM) for 8 h. Expression levels of (**A**) Bcl-xL, Bcl-2, Bak, and Bax and (**B**) cleaved caspase 9, cleaved caspase 3, and cleaved PARP were examined by Western blot (n=4). Untreated cells were used as control.

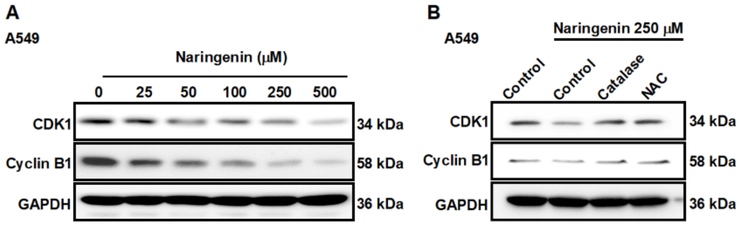


**Figure S6. Naringenin promoted cell cycle arrest through ROS production in A549 cancer cells.** (**A**) A549 cells were treated with naringenin (25-500 μM) for 6 h. CDK1 and cyclin B1 protein expression was examined by Western blot (n=4). (**B**) A549 cells were pretreated with ROS scavengers, catalase (50 U/mL) and NAC (1 mM), for 1 h and then treated with naringenin (250 μM) for 6 h. Protein expression levels of CDK1 and cyclin B1 were examined by Western blot (n=4). Untreated cells were used as control.

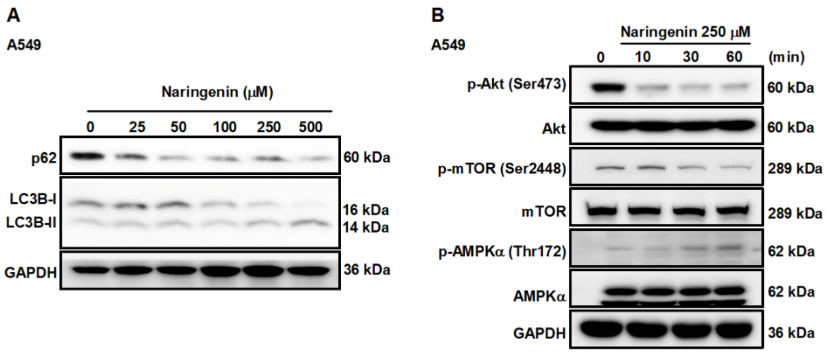


**Figure S7. Naringenin promoted autophagy in A549 cells.** (**A**) A549 cells were treated with naringenin (25-500 μM) for 6 h. Expression of p62 protein and LC3II/LC3I ratio was examined by Western blot (n=4). (**B**) A549 cells were treated with naringenin (250 μM) for the indicated times, after which phosphorylation of Akt, mTOR, and AMPKα was examined by Western blot (n=4). Untreated cells were used as control.

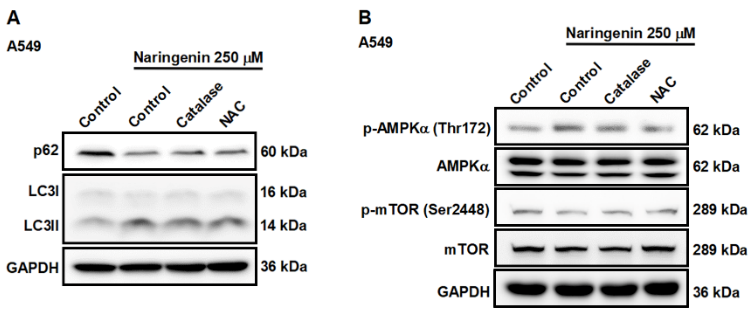


**Figure S8. Naringenin promoted autophagy via ROS production in A549 cells.** (**A**) A549 cells were pretreated with ROS scavengers, catalase (50 U/mL) or NAC (1 mM), for 1 h and then treated with naringenin (250 μM) for 6 h. Expression of p62 protein and LC3II/LC3I ratio was examined by Western blot (n=4). (**B**) A549 cells were pretreated with ROS scavengers for 1 h and then treated with naringenin (250 μM) for 1 h. Phosphorylation of AMPKα and mTOR was examined by Western blot (n=4). Untreated cells were used as control.
